# Analysis of Co-Channel Coexistence Mitigation Methods Applied to IEEE 802.11p and 5G NR-V2X Sidelink

**DOI:** 10.3390/s23094337

**Published:** 2023-04-27

**Authors:** Zhuofei Wu, Stefania Bartoletti, Vincent Martinez, Vittorio Todisco, Alessandro Bazzi

**Affiliations:** 1 College of Computer Science and Technology, Harbin Engineering University, Harbin 150001, China; wzfhrb@hrbeu.edu.cn; 2 Wireless Laboratory (WiLab), National Inter-University Consortium for Telecommunications (CNIT), 40126 Bologna, Italy; 3 Department of Electrical, Electronic and Information Engineering “Guglielmo Marconi” (DEI), University of Bologna, 40126 Bologna, Italy; 4 Department of Electrical Engineering (DEE), University of Rome Tor Vergata, 00133 Rome, Italy; 5 NXP Semiconductors, 31023 Toulouse, France

**Keywords:** connected vehicle, IEEE 802.11p, IEEE 802.11bd, 5G-NR V2X, co-channel coexistence, simulation

## Abstract

Direct communication between vehicles and surrounding objects, called vehicle-to-everything (V2X), is ready for the market and promises to raise the level of safety and comfort while driving. To this aim, specific bands have been reserved in some countries worldwide and different wireless technologies have been developed; however, these are not interoperable. Recently, the issue of co-channel coexistence has been raised, leading the European Telecommunications Standards Institute (ETSI) to propose a number of solutions, called mitigation methods, for the coexistence of the IEEE 802.11p based ITS-G5 and the 3GPP fourth generation (4G) long term evolution (LTE)-V2X sidelink. In this work, several of the envisioned alternatives are investigated when adapted to the coexistence of the IEEE 802.11p with its enhancement IEEE 802.11bd and the latest 3GPP standards, i.e., the fifth generation (5G) new radio (NR)-V2X. The results, obtained through an open-source simulator that is shared with the research community for the evaluation of additional proposals, show that the methods called A and C, which require modifications to the standards, improve the transmission range of one or both systems without affecting the other, at least in low-density scenarios.

## 1. Introduction

Transportation is entering the new era of cooperative, connected and automated mobility (CCAM), with vehicles largely controlled by advanced driver assistance systems (ADAS) and exchanging messages through direct vehicle-to-everything (V2X) wireless communications. Focusing on connectivity, two families of radio access technology (RAT) standards are available today, one based on IEEE 802.11p (and its enhancement IEEE 802.11bd) and the other belonging to the 3GPP cellular ecosystem, including the fourth generation (4G) long term evolution (LTE)-V2X and the fifth generation (5G) new radio (NR)-V2X sidelink (in the paper we will use abbreviations for the sake of conciseness).

Even if the standards for wireless communications appear mature and have indeed already started to be installed on board the new vehicles [1], one of the issues is the fact that no regulations have been currently set to determine which technology should be used in the limited bandwidth that is allocated to the intelligent transport system (ITS). In many countries worldwide, a spectrum has been reserved for exclusive use for direct V2X communications, mostly around 5.9 GHz [2]. In particular, seven channels of 10 MHz (between 5855 MHz and 5925 MHz) have been reserved in the US since 1999, which was reduced to three in 2021 (between 5895 MHz and 5925 MHz). In the US, the FCC explicitly indicated in 2021 that the three remaining channels will be used for C-V2X; however, the statement is not clear on the timeline and the restriction appears to be not actually enforced. There have been five channels (two for non-safety between 5875 and 5895 MHz and three for safety between 5895 and 5925 MHz) reserved in Europe since 2008, which increased to seven in 2021 (those added, both for safety, are from 5825 to 5845 MHz, although from 5835 to 5845 MHz is subject to urban rail priority). For the moment, the regulations define the use of the channels for cooperative intelligent transport systems (C-ITS), including their applicability for safety and non-safety use cases, but they do not indicate a specific wireless access technology. As a consequence, multiple non-interoperable technologies might share the same channel at the same time in the same area, addressing common or separate use cases, possibly leading to inter-technology interference and reduced performance.

The issue of co-channel coexistence has been therefore considered a priority by European Conference of Postal and Telecommunications Administrations (CEPT) [3], which asked the European Telecommunications Standards Institute (ETSI) to introduce some solutions for the mitigation of inter-technology interference, focusing on the two main wireless technologies available at that time, which are ITS-G5, the ETSI technology based on IEEE 802.11p, and LTE-V2X. A number of proposals, called co-channel coexistence mitigation methods and labeled with letters from A to F, were then defined and analyzed in [4]. All these proposals have advantages and drawbacks, and they all imply modifications to one standard or the other. At the time of writing, the discussion is still ongoing between the involved stakeholders and no decision has been taken.

### 1.1. Related Work

An early investigation [5] focuses on the co-channel interference in the presence of periodic or non-periodic traffic generation; the former applies, for example, when the Society of Automotive Engineers (SAE)’s basic safety messages (BSMs) or the ETSI cooperative awareness messages (CAMs) under specific conditions are assumed, whereas the latter applies, for example, when CAMs are considered in more general cases where the vehicle movements (specifically variations of direction, speed, and acceleration) cause the packet generation interval to vary between 100 ms and 1 s [6,7]. The results show that without any mitigation methods applied in the co-channel coexistence scenario, periodic packet generation is preferable to non-periodic traffic in terms of the packet reception ratio (PRR) performance for both technologies. This is because, with periodic traffic, the LTE-V2X could predict more accurately which resources would be accessible in the near future and mitigate the packet collision between these two technologies. The possible benefit of co-channel coexistence mitigation methods for reducing performance loss is then suggested through early simulations in [8].

Later, as required by the CEPT, the feasibility of co-channel coexistence between ITS-G5 and LTE-V2X was investigated by the ETSI [4] and several solutions (Method A∼F as mentioned above and briefly described in the following) were introduced, which are all based on the superframe structure to separate the two technologies into different time domains. The simulation results together with the sub-work in [9] suggest that “Method A and Method C are the two most promising approaches for co-channel coexistence between ITS-G5 and LTE-V2X”. Following this report, a white paper [10] scrutinizes in detail those proposed methods. After confirming that without any mitigation methods, the performance of both technologies degrades significantly due to mutual interference, the white paper confirms the results conducted in [4] that some variants of A and C are preferable to other methods, considering the performance or the implementation complexity. A simplified version of Method C was also proposed later in [11], showing similar or better performance to the original solution, but with a simpler implementation that does not require the use of what are called superframes (see Section 3).

A comparison of selected methods was also provided in the white paper [12], varying the packet size, packet generation period, and vehicle density. The simulation results show that the large packet size (1000 bytes) and the high-density scenario would degrade the performance, especially for Method A. It also concludes that the simplified version of Method C presented in [11] is “the best all-rounder” solution for co-channel coexistence compared with the others.

Apart from the mentioned papers, a few others discuss coexistence scenarios in which two ITS technologies are allocated in different channels, e.g., with vehicles that use both technologies to transfer and relay packets from one technology to the other [13], or assuming that every vehicle uses two technologies simultaneously [14]. These works assume separate bands for the two technologies and vehicles equipped with both of them, which are assumptions not considered in this paper. In particular, in this paper we assume the following:

- Each vehicle is equipped with one of the two technologies, which are IEEE 802.11p and NR-V2X;
- All stations share the same channel and therefore potentially interfere with each other;
- Without loss of generality, we focus on vehicles only (and not road side units).

### 1.2. Contribution and Innovation of the Paper

In this work, for the first time we investigate the applicability of the same methods to the co-channel coexistence of IEEE 802.11p and NR-V2X, which is the latest standard defined by the 3GPP for connected vehicles. We consequently also used IEEE 802.11bd; as clarified in the following, IEEE 802.11bd adds new features to IEEE 802.11p but shares the same access mechanism, so results would be very similar if IEEE 802.11bd was considered with a similar duration of the transmission. Furthermore, although NR-V2X shares similar principles with LTE-V2X, there are two different characteristics that should be taken into account when looking at the co-channel coexistence with IEEE 802.11p/bd:

- NR-V2X introduces numerology; different numerologies lead to different subcarrier spacing (SCS) and transmission time interval (TTI);
- In NR-V2X, the resource selection process is mainly based on reservations indicated in the control information, rather than on the average measured power over the last time interval.

How much these differences impact the performance of technologies under co-channel coexistence has never been addressed in the previous literature, thus deserving further studies. To fill this gap, we here consider several of the coexistence mitigation methods, namely methods A, B, C, and F, discussing their applicability to the new standard and comparing their performance in realistic scenarios. As will be clarified further, the methods that are not analyzed, i.e., D and E, require the implementation in LTE-V2X or NR-V2X stations of the full IEEE 802.11p stack and are therefore not compliant with the assumption that each station is equipped with only one of the technologies.

As part of the contribution of this work, the results are obtained by specifically modifying the open-source simulator WiLabV2Xsim [15], thus providing a platform for the development and testing of new ideas from any researcher interested in this topic. (The simulator is available at https://github.com/V2Xgithub/WiLabV2Xsim (accessed on 25 April 2023) and will include the mitigation methods in its next release).

Results provide a detailed analysis of the performance of both IEEE 802.11p and NR-V2X under several metrics, in scenarios with variable density and variable proportions of vehicles equipped with either technology, and for both periodic and non-periodic traffic. As it will be shown, Methods A and C appear to be the only two able to show an overall positive impact on the performance of the two technologies, although they also imply modifications to the standards and drawbacks that are further clarified in the following. All results are discussed also in comparison with the case of coexistence between IEEE 802.11p and LTE-V2X, highlighting the few but relevant differences.

### 1.3. Paper Organization

IEEE 802.11p, with its enhancement IEEE 802.11bd, and 5G NR-V2X are summarized in Section 2. Section 3 introduces the six mitigation methods, followed by the simulation settings and results in Section 4. Finally, the paper is concluded in Section 5.

## 2. Radio Access Technologies for Direct V2X

In this section, we briefly recall the main aspects of the two wireless technologies under investigation. Let us recall that both technologies define only the lower layers of the protocol pillar, i.e., mainly the physical (PHY) and medium access control (MAC) layers, corresponding to the access layer of the ETSI C-ITS protocol stack [16].

### 2.1. IEEE 802.11p

IEEE 802.11p (hereafter sometimes shortened as 11p for conciseness) was an amendment published in 2012 and is now part of the IEEE 802.11-2020 standard [17]. In the US, it is part of the wireless access in vehicular environment (WAVE) protocol stack that includes documents from IEEE and SAE, while in Europe it is at the basis of ITS-G5 [18].

At the PHY layer, IEEE 802.11p adopts orthogonal frequency-division multiplexing (OFDM) with 64 subcarriers (including virtual subcarriers) and SCS of 156.25 kHz. Convolutional encoding is applied. The generic transmission uses all the subcarriers with the same modulation and coding scheme (MCS), which corresponds in most cases to quadrature phase shift keying (QPSK) with a coding rate of 1/2.

At the MAC layer, carrier sense multiple access with collision avoidance (CSMA/CA) is used; when a vehicular user equipment (VUE) has a frame to transmit, it senses the channel to check if it is currently being used. If the sensed power is below a given threshold, the VUE assumes that the channel is not being used and transmits its frame. Otherwise, it waits until the channel is released, after which it also performs a random backoff procedure to avoid the risk of starting at the same time as some neighboring VUE.

As an evolution of this standard, at the beginning of 2013, the new amendment IEEE 802.11bd [19] was published (hereafter shortened as 11bd for conciseness). Rather than a new solution, it is a fully backward-compatible enhancement to IEEE 802.11p with the scope to improve range and throughput under specific scenarios [20]. More specifically, it is based on the same access scheme (CSMA/CA) and shares the same IEEE 802.11p preamble, and adds a number of new optional features [20,21]: new MCSs with modulations up to 256-QAM; additional pilot sequences, called midambles, to improve the channel estimation when the mobility is high and the packet length is particularly large [22]; up to three blind packet retransmissions (called blind because they are not related to acknowledgments) [23]; possible use of channel bonding [24]; and mmWave band [25].

Dealing with co-channel coexistence, it is relevant to note that the CSMA/CA protocol in IEEE 802.11p (and IEEE 802.11bd) has an implicit procedure to limit interference to other signal sources, no matter the technology used. It should be noted, however, that the power threshold to assume the channel as busy when the signal cannot be recognized is −65 dBm, which is much higher than what is used when the signal is recognized instead. A frame being sent by another IEEE station can in fact be recognized by the preamble detection, which depends on the specific implementation of the receiver, but can be realistically assumed around −100 dBm. The lower power level implies a reduction in the probability of collisions due to hidden terminals, as discussed in detail in [11].

### 2.2. NR-V2X

Sidelink was first introduced to address public safety and other device-to-device (D2D) proximity services by 3GPP in Release 12, and then specifically tailored for V2X in 2017 with Release 14. In 2020, sidelink was added to the latest 5G NR-V2X.

NR-V2X is based on orthogonal frequency-division multiple access (OFDMA), which implies multi-carrier modulation at the PHY layer and synchronous time–frequency division at the MAC layer. Different options are possible at the PHY layer, indicated as numerology, which implies in the sub-6 GHz bands either 15, 30, or 60 kHz SCS. The number of subcarriers depends on both the SCS and the channel bandwidth. The granularity for resource allocation is given by the TTI in the time domain and the subchannel in the frequency domain. The TTI corresponds to 14 OFDM symbols and therefore lasts 1, 0.5, or 0.25 ms depending on the numerology. The subchannel corresponds to a certain number of groups of 12 subcarriers, which is set by the network.

The use of the channel in NR-V2X is based on the principle of orthogonal resources and resource reservation, and can exploit a mechanism called sensing-based semi-persistent scheduling (SB-SPS). Every time a VUE transmits it also adds information to reserve another resource in the future if needed; the reservation can be made periodically for some time, from which the name semi-persistent is derived. Based on this, the neighboring VUEs with something to transmit consider that some resources are reserved and select different ones. No reservation can be performed before the first frame is transmitted. Details in the procedure can be found, for example, in [15,26].

Differently from CSMA/CA, the procedure defined for NR-V2X cannot prevent collisions with signals that belong to a different technology. It can be also observed that, even if the resource allocation procedure is a derivation of the SB-SPS of LTE-V2X, it is substantially different in NR-V2X. In LTE-V2X, in fact, the identification of reserved resources is mainly performed based on the average power measured in the last 1 s. In the case of LTE-V2X, periodic transmissions performed by IEEE 802.11p VUEs could lead to the identification of a reservation by cellular VUEs and, as a consequence, reduce the risk of collision. This aspect is further detailed for example in [5,12]. In contrast, the NR-V2X sidelink makes decisions based on control information that is not available within IEEE 802.11p signals.

## 3. Co-Channel Coexistence Mitigation Methods

As already anticipated, a number of co-channel coexistence mitigation methods, indicated by the letters from A to F have been defined in [4]. Hereafter, the main common principles and a brief description of the various proposals are provided. To simplify the reading, rather than just using the letters, we will give short names to the methods. A summary comparison of the methods is provided in Table 1. Since each of the methods adds new or modified messages, a summary of them is provided in Table 2.

In the following, the vehicles equipped with IEEE 802.11p are denoted as $V_{11p}$ and those equipped with NR-V2X are denoted as $V_{\mathrm{NR}}$.

### 3.1. Superframe and Slot

**Figure 1:**
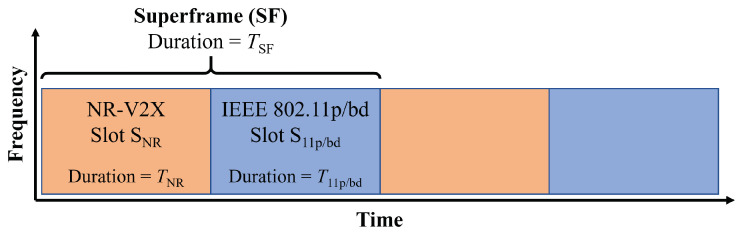
Illustration of the superframe used in the mitigation methods. Each superframe consists of two parts: the NR-V2X slot used by $V_{\mathrm{NR}}$ and the IEEE 802.11p/bd slot used by $V_{11p}$.

All of the mitigation methods mentioned in this paper are based on the concepts of superframeand slot. The superframe SF is a time interval consisting of two slots, one reserved for NR-V2X (hereafter denoted as $S_{\mathrm{NR}}$), and the other for IEEE 802.11p (hereafter denoted as $S_{11p/\mathrm{bd}}$). These concepts are exemplified in Figure 1, where the $T_{\mathrm{SF}}$, $T_{\mathrm{NR}}$, and $T_{11p/\mathrm{bd}}$ stand for the durations of the corresponding intervals.

The superframe duration is in all the cases fixed. The slot duration can instead be fixed, hereafter called static superframe settings, or variable, hereafter called dynamic superframe settings. The dynamic solution is preferable since it can better cope with variable distributions of the two technologies among the VUEs, but it introduces more complexity. In addition, the slots can be indicated by an external entity, called hereafter controlled superframe settings, or estimated independently by each node, called hereafter autonomous superframe settings. The use of an external entity can simplify the methods since all nodes then have the same settings, but also means that road side units (RSUs) are required and additional vehicle-to-infrastructure (V2I) communication protocols need to be defined.

It should be noted that superframes and slots are relatively easy to implement in NR-V2X, since it is already a time-slotted system and already foresees the possibility of having pools of available resources as a subset of all resources. In contrast, IEEE 802.11p is based on completely asynchronous access to the channel.

### 3.2. $M_{A}$-Time-Split

**Figure 2:**
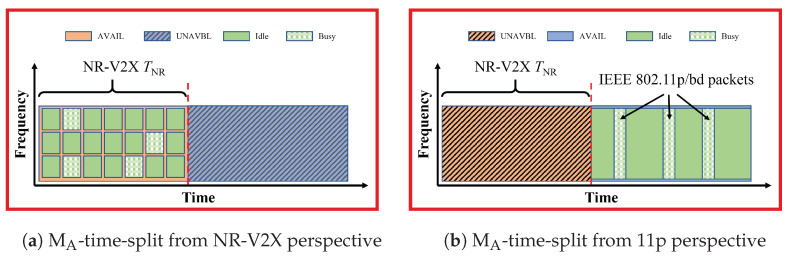
Illustration of $M_{A}$-time-split, both vehicles $V_{\mathrm{NR}}$ and $V_{11p}$ are synchronized and know the structure of the superframe. Each type of vehicle could only transmit packets on their own slot $S_{\mathrm{NR}}$ or $S_{11p/\mathrm{bd}}$. The subfigure (**a**) is from the $V_{\mathrm{NR}}$ perspective, and the subfigure (**b**) is from the $V_{11p}$ perspective. In this and the following figures, a vertical dashed red line is added to separate the NR-V2X slot from the 11p/bd slot.

***Brief description.*** With $M_{A}$-time-split, both types of vehicles $V_{\mathrm{NR}}$ and $V_{11p}$ are supposed to be synchronized to the superframes, with knowledge of $S_{\mathrm{NR}}$ and $S_{11p/\mathrm{bd}}$. This means that the vehicles are all synchronized and informed of the adopted settings. The VUEs are allowed to transmit only in their respective slots, as shown in Figure 2.

***Enhanced version.*** One issue arising with $M_{A}$-time-split is the so-called *channel rush problem* [10]; all $V_{11p}$ who have new messages to transmit during $S_{\mathrm{NR}}$ would hold on, and they all would start the backoff procedure immediately when the time goes into the $S_{11p/\mathrm{bd}}$, leading to a higher probability of collisions at the beginning of $S_{11p/\mathrm{bd}}$. To solve this issue, enhanced $M_{A}$-time-split (hereafter e$M_{A}$-time-split) was defined, where $V_{11p}$ always add a certain delay before starting the channel sensing procedure, in a way that uniformly distributes the beginning of transmissions. More details can be found in [10].

***Configurations.*** This method is easily implemented only with static and controlled slot settings. The other options need further work.

***Modifications required.*** This method is easily implemented in NR-V2X through the definition of resource pools, which can include only the TTIs belonging to the $S_{\mathrm{NR}}$. A difference is that it requires modifications to the IEEE 802.11p VUEs, which are normally not synchronized; the $V_{11p}$ need to align to the superframe and assume as busy the slot reserved by NR just like the sensed power was above the threshold. Given that modifications are required anyway for the IEEE 802.11p specifications and that the performance of the $M_{A}$-time-split is substantially worse, only the e$M_{A}$-time-split is considered further.

***Implications of NR instead of LTE.*** Given that each technology has exclusive access to its own slot, the use of NR-V2X instead of LTE-V2X does not impact the inter-technology interference received or generated by IEEE 802.11p.

### 3.3. $M_{B}$-E-Signals

**Figure 3:**
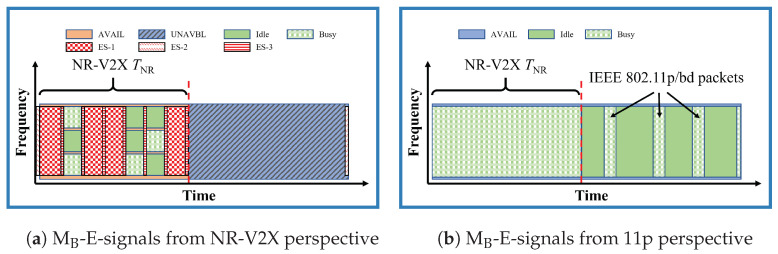
Illustration of $M_{B}$-E-signals, only $V_{\mathrm{NR}}$ know the superframe structure and they would transmit 3 types of energy signal during the idle TTI, just before $S_{\mathrm{NR}}$, and at the 14th OFDM symbol in each TTI to inform $V_{11p}$ the $S_{\mathrm{NR}}$ is busy. The subfigure (**a**) is from the $V_{\mathrm{NR}}$ perspective, and the subfigure (**b**) is from the $V_{11p}$ perspective.

***Brief description.*** In $M_{B}$-E-signals, as shown in Figure 3, only $V_{\mathrm{NR}}$ know the superframe structure, and they can only transmit packets in their own slot $S_{\mathrm{NR}}$. To make $V_{11p}$ aware of the slot reserved for NR, $V_{\mathrm{NR}}$ transmit three types of energy signals (ESs) that do not carry any data:

1. ES-1 is sent during a TTI in $S_{\mathrm{NR}}$ that is not used by any $V_{\mathrm{NR}}$; the signal is transmitted by all $V_{\mathrm{NR}}$ that do not transmit data and sense the TTI as idle;
2. ES-2 is sent just before the slot $S_{\mathrm{NR}}$ to avoid $V_{11p}$ initiating a transmission at the end of the last part of $S_{11p/\mathrm{bd}}$, which would then partially overlap with the following $S_{\mathrm{NR}}$; the signal is transmitted by all $V_{\mathrm{NR}}$ that sense the channel idle during that interval;
3. ES-3 is sent during the last OFDM symbol of each TTI in $S_{\mathrm{NR}}$ by all $V_{\mathrm{NR}}$ that transmitted during that TTI, with the scope to avoid $V_{11p}$, assuming that the channel is idle during that gap.

When $V_{11p}$ sense the ESs, they assume that the channel is busy and defer any access to the channel; this is automatically performed by CSMA/CA, without the need to change the protocol.

***Configurations.*** The energy signals can be implemented with variable slot settings, but this appears possible only if they are controlled, because all NR stations should have the same view of the slots.

***Modifications required.*** The NR stations need to transmit the ESs (which are new, as reported in Table 2). The only modification required in IEEE 802.11p in order to correctly detect the ESs, is to reduce the clear channel assessment (CCA) threshold of $V_{11p}$ (above which the channel is assumed busy) from −65 dBm to −85 dBm; otherwise, the probability that an ES is ignored is too high. This modification would require further studies, since it may also increase the probability of channel busy detection due to spurious emissions, normally ignored with the current threshold.

***Implications of NR instead of LTE.*** The use of NR-V2X instead of LTE-V2X implies that when the numerology is higher than 0, which means that the the duration of the TTI and the OFDM symbols are reduced (please refer to the first three columns of Table 3), the time duration of both ES-1 and ES-3 is shorter.

### 3.4. $M_{C}$-Preamble

**Figure 4:**
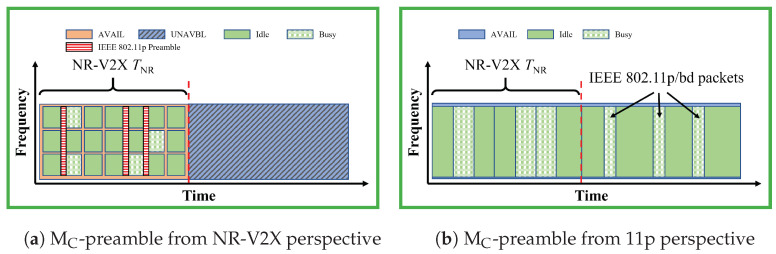
Illustration of $M_{C}$-preamble. Only $V_{\mathrm{NR}}$ know the superframe structure. An IEEE 802.11p preamble is added at the head of each NR-V2X packet to inform $V_{11p}$ that the channel is busy in slot $S_{\mathrm{NR}}$. The subfigure (**a**) is from the $V_{\mathrm{NR}}$ perspective, and the subfigure (**b**) is from the $V_{11p}$ perspective.

***Brief description.*** In the $M_{C}$-preamble, the superframes are known only by $V_{\mathrm{NR}}$. With this method, as illustrated in Figure 4, an IEEE 802.11p preamble is added at the beginning of each NR-V2X transmission to inform the $V_{11p}$ that the channel is occupied for a given duration (up to 10 ms using the standard preamble). Please note that the “IEEE 802.11p preamble” here is actually the “preamble field” + “signal field” in the physical layer protocol data unit (PPDU) format; the information of the packet length is included in the signal field [17]. The preamble uses a 10 MHz channel and lasts 40 µs.

For the transmission of the preamble, the gap before each transmission and the first OFDM symbol can be used; the first OFDM symbol, in fact, carries a copy of the second symbol and is used for automatic gain control (AGC), thus using it does not reduce the data rate of NR.

The IEEE 802.11p preambles added to each NR-V2X packet are identical. Thus, given the OFDM properties, multiple preambles sent in the same TTI by different $V_{\mathrm{NR}}$ are treated by the generic receiver as a single signal affected by multi-path.

***Configurations.*** This method can be implemented with a static or dynamic slot configuration. As discussed in [10], the static configuration performs similarly to the e$M_{A}$-time-split, thus in this paper we only consider the dynamic configuration (hereafter called d$M_{C}$-preamble) where each of the $V_{\mathrm{NR}}$ estimates the NR slot $T_{\mathrm{NR}}$ locally, based on the perceived technology percentage. The perceived technology percentage can be derived by $V_{\mathrm{NR}}$ for example from the equation

(1) $${\mathrm{Tech}}_{\%}=\frac{{\mathrm{CBR}}_{\mathrm{NR}}}{{\mathrm{CBR}}_{\mathrm{NR}+11p}}$$

where ${\mathrm{CBR}}_{\mathrm{NR}}$ is the channel occupation measured only considering the NR-V2X signals, and ${\mathrm{CBR}}_{\mathrm{NR}+11p}$ is measured considering also the other signals, as suggested in [4] and used hereafter.

The PHY header always indicates 1 TTI, which is dependent on the numerology, to cope with different possible estimations of the slot duration. The time assigned to the $S_{\mathrm{NR}}$ and $S_{11p/\mathrm{bd}}$ cannot be less than 5ms [4].

***Modifications required.*** This method does not require any change to IEEE 802.11p. NR needs instead to be modified to include the IEEE 802.11p preamble (reported in Table 2) and the technology proportion estimation. At the same time, it can be noted that the preamble is always the same, thus it can be implemented as a fixed sequence of IQ samples, and the technology proportion may not be required if a static or controlled configuration is used.

***Implications of NR instead of LTE.*** It is worth noting that the preamble needs at least a 40µs time space to be inserted. As shown in Table 3, the time period that can be inserted is different depending on the numerology. With numerology 0, which corresponds to the LTE, it is possible to transmit the preamble within either the gap or the first symbol. With numerology 1, both of them (71.4 µs in total) need to be used. Finally, with numerology 2 one additional symbol is needed, with an impact on the NR data rate.

***The variant *$M_{C}$*-preamble-no-SF. ***$M_{C}$-preamble-no-SF, firstly proposed in [11], is similar to $M_{C}$-preamble but without the superframe and the slot notations. With this method, the whole time domain is available for $V_{\mathrm{NR}}$ and $V_{11p}$. With this variant, fewer modifications are required in $V_{\mathrm{NR}}$; it in fact only requires that the preamble of IEEE 802.11p is added at the beginning of NR-V2X packets. With this variant, the superframe is not present anymore and therefore there is no distinction between dynamic, static, and semi-static configuration, and the solution is inherently fully distributed.

### 3.5. $M_{F}$-CTS-to-Self

**Figure 5:**
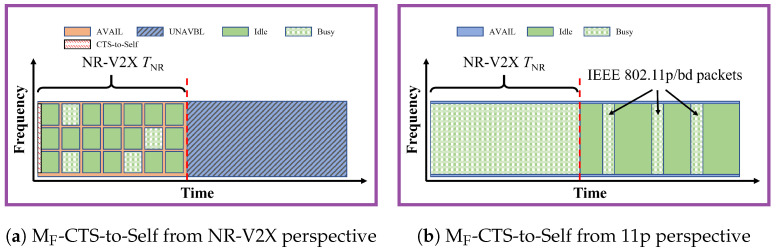
Illustration of $M_{F}$-CTS-to-Self. Only $V_{\mathrm{NR}}$ know the superframe structure. At the beginning of each $S_{\mathrm{NR}}$, a chosen $V_{\mathrm{NR}}$ would broadcast a CTS-To-Self message to reserve the resources from $V_{11p}$. The subfigure (**a**) is from the $V_{\mathrm{NR}}$ perspective, and the subfigure (**b**) is from the $V_{11p}$ perspective.

***Brief description.*** In $M_{F}$-CTS-to-Self, as illustrated in Figure 5, the superframe structure is again only known by $V_{\mathrm{NR}}$. At the beginning of each $S_{\mathrm{NR}}$, a Clear-To-Send-To-Self (CTS-To-Self) message is sent by some of the $V_{\mathrm{NR}}$ to inform the $V_{11p}$ that the channel will be reserved. The CTS-To-Self message is specified in the MAC layer of IEEE 802.11 for channel resource reservation to avoid the hidden terminal problem. The channel could be reserved for up to 32 ms by the CTS-To-Self message.

When the $V_{11p}$ receive the CTS-To-Self message, they set a parameter named network allocation vector (NAV) to mark that the channel is busy during the indicated time interval, regardless of the actual sensed status of the channel.

**Figure 6:**
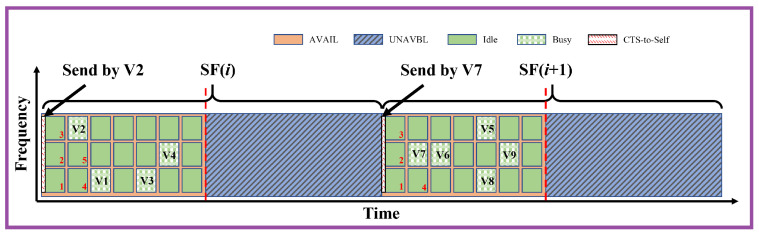
Procedure of choosing the CTS-To-Self sender of $M_{F}$-CTS-to-Self. $V_{2}$ has a resource segment in the SF(*i*), and no resources are reserved by other vehicles before it, hence the CTS-To-Self in SF(*i*) would be transmitted by $V_{2}$ at the beginning of SF(*i*). Following the same procedure, $V_{7}$ is chosen to send the CTS-To-Self message at SF(*i*+1). The red number in the figure is the resource index ordered in the frequency and time domains.

The reservation information of the CTS-To-Self message is included in the DATA field, thus the scrambling operation in the PHY layer would lead to different signals varying with the transmitter. This implies that multiple messages sent simultaneously from multiple sources collide. To reduce this issue, only selected nodes transmit the CTS-To-Self message. In general, there may be multiple possible rules to determine which $V_{\mathrm{NR}}$ are selected. Consistently with what is proposed in [4], in this paper the vehicles that have resources in the current superframe and do not sense anyone reserving resources before it or during the same slot with lower frequency will send the CTS-To-Self message. An example of this approach is given in Figure 6.

***Configurations.*** Given that only part of the stations transmit the CTS-To-Self, how to apply it in a dynamic configuration is not clear. Thus, only the static and autonomous superframe setting is investigated in this paper.

***Modifications required.*** VUE with NR-V2X should be changed to be able to send the CTS-To-Self message (reported in Table 2) and make decisions about which vehicle needs to send the CTS-To-Self message, while VUE with IEEE 802.11p should be able to recognize the CTS-To-Self message and set the NAV accordingly.

***Implications of NR instead of LTE.*** The use of NR-V2X instead of LTE-V2X with numerology above 0 implies shorter messages. This is, however, not expected to impact relevantly on the inter-technology interference received or generated by IEEE 802.11p stations.

### 3.6. Methods with Channel Reservation

The last two methods, which we call $M_{D}$-reservation and $M_{E}$-reservation-&-preamble, are here briefly described for completeness but not considered in the following. As already anticipated, in fact, these two methods require that the $V_{\mathrm{NR}}$ use CSMA/CA following the IEEE 802.11p standard to gain the access to the channel.

In particular, the $M_{D}$-reservation and $M_{E}$-reservation-&-preamble rely on a new message (see Table 2), which carries resources reservation information and can be used by both $V_{\mathrm{NR}}$ (equipped with IEEE 802.11p transceiver) and $V_{11p}$ to reserve the channel for a certain time interval. $V_{\mathrm{NR}}$ cannot access the channel before they reserve resources by accessing the channel through CSMA/CA and send the new reservation message. This implies also a modification of the legacy IEEE 802.11p to be able to read the same message.

**Figure 7:**
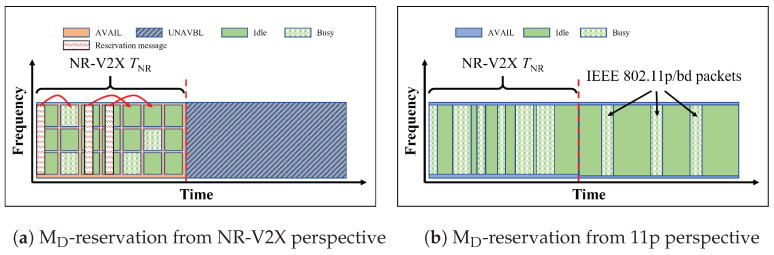
Illustration of $M_{D}$-reservation. Only $V_{\mathrm{NR}}$ know the superframe structure. Before transmitting the NR-V2X packet, $V_{\mathrm{NR}}$ would broadcast an IEEE 802.11p type reservation message to reserve resources. The subfigure (**a**) is from the $V_{\mathrm{NR}}$ perspective, and the subfigure (**b**) is from the $V_{11p}$ perspective.

In $M_{D}$-reservation, only $V_{\mathrm{NR}}$ knows the superframe structure. As shown in Figure 7, in the slot $S_{\mathrm{NR}}$, before transmitting an NR-V2X packet, $V_{\mathrm{NR}}$ broadcast an IEEE 802.11p type reservation message to let $V_{11p}$ and other $V_{\mathrm{NR}}$ know that during the following time interval the channel is reserved.

**Figure 8:**
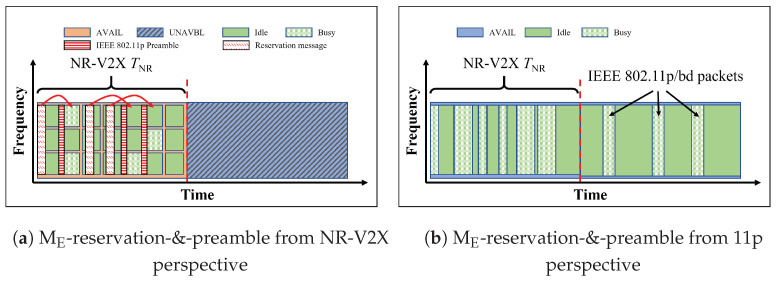
Illustration of $M_{E}$-reservation-&-preamble. It is a combination of methods C and D. Only $V_{\mathrm{NR}}$ know the superframe structure and they would use both the IEEE 802.11p preamble and reservation message to reserve resources. The subfigure (**a**) is from the $V_{\mathrm{NR}}$ perspective, and the subfigure (**b**) is from the $V_{11p}$ perspective.

As shown in Figure 8, $M_{E}$-reservation-&-preamble combines the use of the reservation messages (as $M_{D}$-reservation) and the IEEE 802.11p preamble insertion (as $M_{C}$-preamble) to further improve the ability of $V_{11p}$ to avoid colliding with $V_{\mathrm{NR}}$.

## 4. Simulations and Results

We investigate the co-channel coexistence performance of IEEE 802.11p and NR-V2X, possibly amended with the mitigation methods e$M_{A}$-time-split, $M_{B}$-E-signals, d$M_{C}$-preamble, $M_{C}$-preamble-no-SF, and $M_{F}$-CTS-to-Self. Results are provided using the open-source V2X simulator WiLabV2Xsim [15].

### 4.1. Simulation Settings and Results Format

The main simulation settings are summarized in Table 4. A highway scenario is assumed, with vehicles uniformly distributed over six lanes (three per direction) with variable density. Each vehicle is equipped with one of the two technologies, selected randomly with different proportions. Both a balanced distribution, with 50–50% IEEE 802.11p and NR-V2X, and an unbalanced distribution, with 33–66% are considered. In the case of unbalanced, the case with a majority of VUEs equipped with IEEE 802.11p is denoted as the *more-$V_{11p}$ scenario* and the one with a majority of VUEs equipped with NR-V2X is denoted as the *more-$V_{\mathrm{NR}}$ scenario*. It can be noted that the 33–66% distribution is far from the worst case and allows us to investigate a quite common situation under normal traffic where the local distribution of technologies cannot be controlled and would be presumably highly variable.

Packets of 350 bytes are assumed, which is consistent with the average CAM packet size observed in [28]. The packets are either generated periodically every 100 ms (the highest frequency for CAM messages) or following the CAM rules.

Regarding the propagation, the modified version of the ECC Report 68 path-loss model detailed in [27] is used in this paper, which is characterized by a path-loss exponent of 3.3 and range in good agreement with on-field measurements (the same model is used for both technologies). Log-normal shadowing is also considered, with a variance of 3 dB and decorrelation distance of 25 m. Fast fading is taken into account by the PHY layer abstraction as detailed in [29]. It can be observed that the studied scenario is 2-D; different models, such as those proposed in [30,31,32] could be used when a 3-D scenario was investigated in the future.

Commonly used settings are adopted for MAC and PHY in the two technologies. The MCS in NR-V2X is chosen as the lowest possible (most reliable) for the given channel. Furthermore, blind retransmissions are not enabled in NR-V2X since this would strongly and unfairly affect the impact of coexistence and methods; investigating the impact when blind retransmissions are enabled is left for future work.

When used, the superframe duration is set to 25ms, which is the middle value between those indicated in [4] (10, 25, or 50 ms). When a static configuration is assumed, the slot time is fixed to $T_{\mathrm{NR}}$ = 13ms and $T_{11p/\mathrm{bd}}$ = 12ms, regardless of the balanced or unbalanced vehicle distributions; in real scenarios, in fact, the technology distribution would likely change in time and space and such variability could not be caught in the static configuration. It is therefore relevant to take such inaccuracy into account.

Since the investigated mechanisms impact the channel occupation measurement (as shown in [12]) and therefore alter the way congestion control mechanisms work in either technology, congestion control is not used in this paper; the impact of congestion control and its improvement in the coexistence scenarios is left for future work.

**Figure 9:**
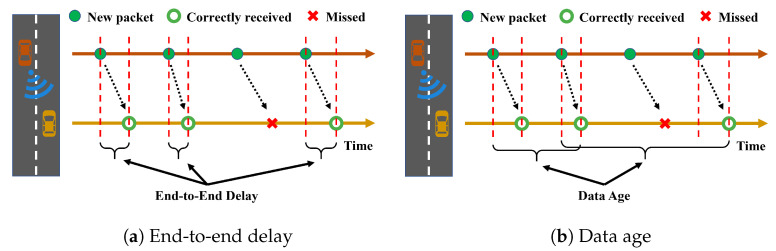
Exemplification of (**a**) end-to-end delay and (**b**) data age.

Three metrics are used to compare the performance of each mitigation method:

(1) ***Transmission range (TR)***: The maximum distance to have an average *PRR* higher than 0.9, where the PRR at a given distance (with granularity 50 m) is defined as the ratio between the number of vehicles receiving the packet correctly at the given distance from the receiver and the total number of target vehicles at the same distance;
(2) ***End-to-end delay (EED)***: The time interval between the generation time of a packet at the transmitter and its reception time at the receiver. As shown in Figure 9a, only correct reception contributes to this metric;
(3) ***Data age (DA)***: The time interval between the generation of a packet that is correctly received and the time when the next correct reception occurs (from the same source to the same receiver); this time is also equal to the time between two consecutive received packets generated from the same transmitter plus the EED of the first packet. As shown in Figure 9b, errors increase the DA, which therefore takes into account the correlation between errors.

In all the plots, the gray lines provide benchmarks where no methods are used. In particular, the dotted gray lines correspond to results with all vehicles equipped with the same technology, called the *Only-1-tech* scenario in the following. The dashed gray lines correspond to the *No-method* scenario, where the two technologies coexist with no mitigation methods. The colored lines correspond to the performance when either of the mitigation methods listed in Table 1 are used (the corresponding colors are used to frame Figure 2, Figure 3, Figure 4, Figure 5 and Figure 6).

### 4.2. Results with Balanced Technology Distribution

**Figure 10:**
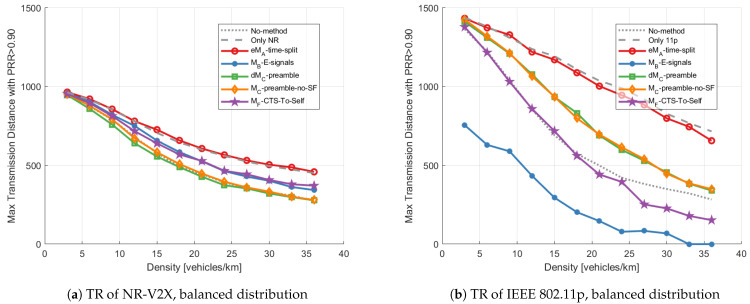
Transmission range vs. density applying different mitigation methods with the balanced distribution; (**a**) shows the transmission range of NR-V2X, and (**b**) shows the transmission range of IEEE 802.11p.

***Transmission range:*** Figure 10 illustrates the transmission range with a balanced number of $V_{\mathrm{NR}}$ and $V_{11p}$ running on the road. As the red lines and the gray dashed lines show, the e$M_{A}$-time-split has a similar performance compared with the Only-1-tech scenario. This is reasonable because the e$M_{A}$-time-split has half of the accessible resources and half of the nodes in each technology compared to Only-1-tech.

Compared with the No-method results, d$M_{C}$-preamble and $M_{C}$-preamble-no-SF improve the transmission range of IEEE 802.11p (Figure 10b), while not reducing the performance of NR-V2X (Figure 10a). Because the use of the preamble added to NR-V2X signals, the $V_{11p}$ can assess the channel status more accurately, which reduces the packet collision probability compared to the No-method test. It can be noted that the improvement obtained is lower than with LTE-V2X (as shown in [12] and consistently with the discussion in [11]). This is due to a shorter slot in NR-V2X, which implies a higher probability that a new transmission from NR-V2X starts during the transmission of the generic IEEE 802.11p packet. Similarly to the results observed in [12] concerning LTE-V2X, $M_{C}$-preamble-no-SF never performs worse than d$M_{C}$-preamble also in the case of NR-V2X, despite its simpler implementation. Rather, $M_{C}$-preamble-no-SF even slightly outperforms d$M_{C}$-preamble from the NR-V2X perspective. To understand this behavior, it should be noted that with $M_{C}$-preamble-no-SF the $V_{\mathrm{NR}}$ have access to all resources instead of part of them; this makes the interference generated and received by $V_{\mathrm{NR}}$ spread in time over a longer interval, but with the same average intensity.

$M_{B}$-E-signals and $M_{F}$-CTS-to-Self improve the transmission range of $V_{\mathrm{NR}}$ compared to d$M_{C}$-preamble and $M_{C}$-preamble-no-SF, as these methods can deny $V_{11p}$ access to the NR slot more effectively than the other two, either through the energy signals or the CTS-To-Self messages. However, the performance of $V_{11p}$ with $M_{B}$-E-signals is reduced because the used energy signals do not have a sensing process before accessing the channel, and therefore strongly increase the probability of collisions with 11p packets. With $M_{F}$-CTS-to-Self, the performance of $V_{11p}$ are reduced under high density due to the fact that not all of the $V_{11p}$ always receive the CTS-To-Self messages; therefore they may access the channel during $S_{\mathrm{NR}}$ and be interfered with by NR-V2X transmissions.

**Figure 11:**
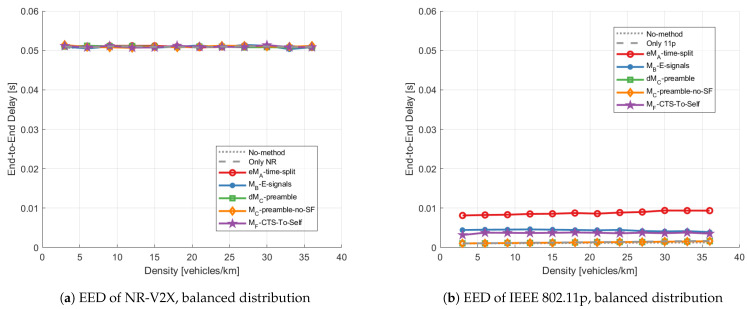
Average End-to-End Delay within 300 m vs. density applying different mitigation methods with balanced distribution scenario; (**a**) shows the average EED of NR-V2X packet, and (**b**) shows the average EED of IEEE 802.11p packet.

***End-to-end delay:*** The results in terms of average EED for links within 300 m are plotted in Figure 11. Looking at Figure 11a, the average EED of NR-V2X is not influenced by the mitigation methods, as already highlighted for LTE-V2X in [12]. NR-V2X is in fact designed to respect a certain delay budget, and the average EED is equal to the mean between the minimum and maximum allowed delay.

As shown in Figure 11b, the EED changes for IEEE 802.11p. In particular, the e$M_{A}$-time-split significantly increases the average EED of IEEE 802.11p packets, because all packets generated by $V_{11p}$ during the $S_{\mathrm{NR}}$ are delayed until the next $S_{11p/\mathrm{bd}}$. The additional average delay introduced by the e$M_{A}$-time-split depends on the superframe structure and is not different when comparing LTE-V2X and NR-V2X. $M_{B}$-E-signals and $M_{F}$-CTS-to-Self also cause an increased delay for the same reason, although this is lower. This is due to the fact that there are cases where the $V_{11p}$ do not measure the energy signals or do not receive the CTS-To-Self messages and thus transmit during $S_{\mathrm{NR}}$. d$M_{C}$-preamble and $M_{C}$-preamble-no-SF perform the same as the No-method and Only-1-tech method, since they do not alter the access mechanism of IEEE 802.11p. It is worth noting that the increased EED should be acceptable for Day-1 type applications that share vehicle status, such as CAMs. The common feature of Day-1 applications is in fact that they are relatively simple, human driver-based and free of driving control [33]. In this perspective, the Day-1 applications would only show warning messages and make the drivers aware of the environment. Considering the reaction time of drivers, the slight increase in EED (no more than 0.01 s) should be acceptable for the Day-1 applications [34]. The additional delay might instead become a problem for Day-2 delay-sensitive applications that share the vehicle sensor data, such as collective perception messages (CPMs) or maneuver coordination messages (MCMs).

**Figure 12:**
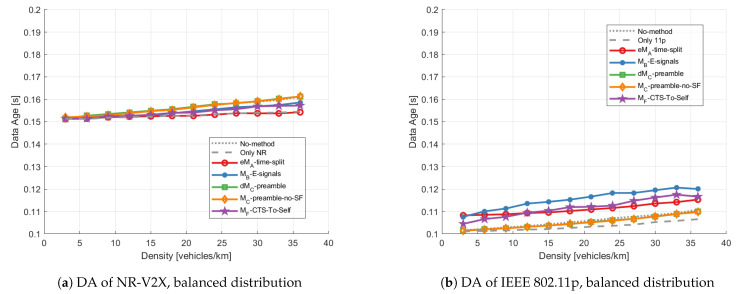
Average data age within 500 m vs. density applying different mitigation methods with the balanced distribution: (**a**) shows the average DA of NR-V2X, and (**b**) shows the average DA of IEEE 802.11p.

***Data age:*** Figure 12 shows the data age for links within 500 m. As shown in both subfigures, the DA slightly increases with a higher density. The preferable method is marginally e$M_{A}$-time-split from the viewpoint of NR-V2X, and d$M_{C}$-preamble and $M_{C}$-preamble-no-SF from that of IEEE 802.11p. Only d$M_{C}$-preamble and $M_{C}$-preamble-no-SF do not perform worse than the No-method (dotted gray line) for both IEEE 802.11p and NR-V2X technologies. The other mitigation methods reduce the DA of NR-V2X but cause a DA of IEEE 802.11p larger than with the No-method scenario, with $M_{B}$-E-signals performing worst.

### 4.3. Results with Unbalanced Technology Distribution

**Figure 13:**
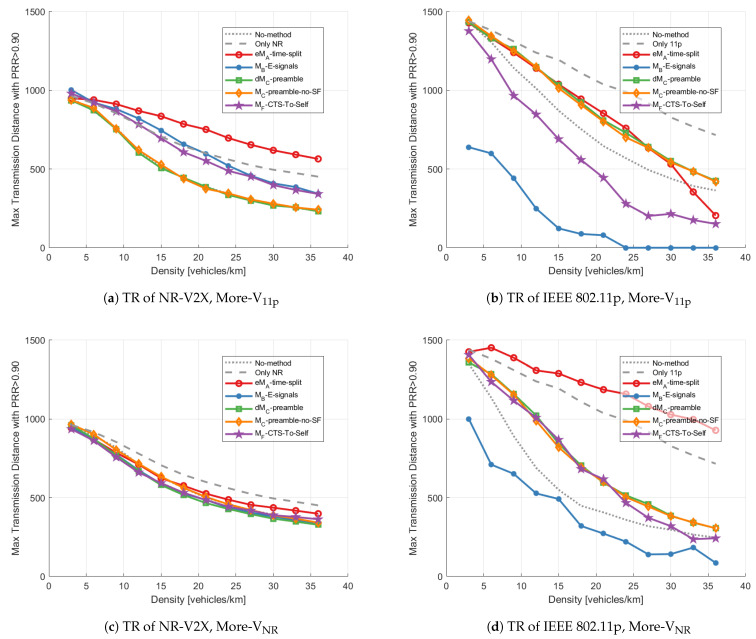
Transmission range vs. density applying different mitigation methods with the unbalanced distribution: (**a**) TR of NR-V2X with More-$V_{11p}$, (**b**) TR of IEEE 802.11p with More-$V_{11p}$, (**c**) TR of NR-V2X with More-$V_{\mathrm{NR}}$, (**d**) TR of IEEE 802.11p with More-$V_{\mathrm{NR}}$

***Transmission range:*** The TR with unbalanced technology distribution is plotted in Figure 13, both for More-$V_{11p}$ (Figure 13a,b) and More-$V_{\mathrm{NR}}$ (Figure 13c,d). When we focus on the TR of NR-V2X (see Figure 13a,c), e$M_{A}$-time-split provides better performance than all the other methods, even with heavier NR-V2X traffic (More-$V_{\mathrm{NR}}$). $M_{C}$-preamble-no-SF is the only method that maintains similar performance in the two scenarios. It also performs slightly better than the d$M_{C}$-preamble in the More-$V_{\mathrm{NR}}$ scenario, because the d$M_{C}$-preamble has to reserve at least 5ms for $S_{11p/\mathrm{bd}}$ while $M_{C}$-preamble-no-SF does not have this limitation.

Observing Figure 13b,d, d$M_{C}$-preamble and $M_{C}$-preamble-no-SF improve the TR of IEEE 802.11p compared to the No-method results and outperform the other methods if we exclude the e$M_{A}$-time-split method. In the case of e$M_{A}$-time-split, the resources reserved for each technology are always approximately the 50% (we are assuming a static configuration) and therefore the average number of IEEE 802.11p transmissions attempted in the $S_{11p}$ is lower than in the balanced case with More-$V_{\mathrm{NR}}$ and higher with More-$V_{11p}$. This implies that under the More-$V_{\mathrm{NR}}$ scenario there are on average fewer 11p transmissions in the same interval compared to the balanced case, implying a lower collision probability. When there are relatively more $V_{11p}$, it means that more transmissions are performed on average in the same time interval and therefore the packet collision probability increases, in turn reducing the transmission range.

Furthermore, in the unbalanced case, $M_{B}$-E-signals for any vehicle density and $M_{F}$-CTS-to-Self for high vehicle density imply lower TR for IEEE 802.11p than with the No-method test.

***End-to-end delay:*** Results of average EED within 300 m with unbalanced technology percentages are very similar to the balanced case, and are therefore not shown.

**Figure 14:**
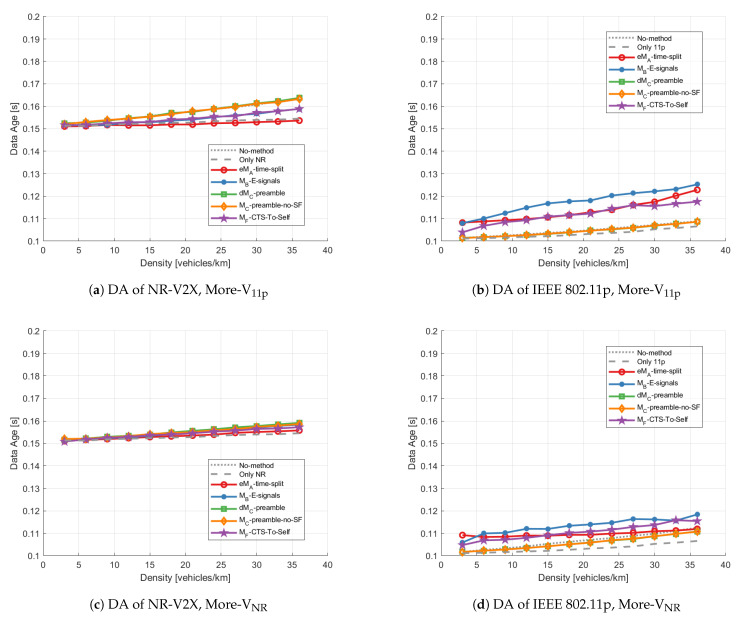
Average data age vs. density within 500 m applying different mitigation methods with the unbalanced distribution: (**a**) DA of NR-V2X with More-$V_{11p}$, (**b**) DA of IEEE 802.11p with More-$V_{11p}$, (**c**) DA of NR-V2X with More-$V_{\mathrm{NR}}$, (**d**) DA of IEEE 802.11p with More-$V_{\mathrm{NR}}$

***Data age:*** Figure 14 shows the results of DA for links within 500 m in the unbalanced cases. Similar conclusions to the balanced case can be inferred. Concerning NR, as shown in Figure 14a,c, the No-method and the Only-NR are the upper and lower bounds of all mitigation methods, which means that the lower number of vehicles are equipped with IEEE 802.11p, the better DA is for $V_{\mathrm{NR}}$. From the 11p perspective (Figure 14b,d), overall, only d$M_{C}$-preamble and $M_{C}$-preamble-no-SF do not perform worse than the No-method scenario (dotted gray line) for both technologies.

### 4.4. Impact of Periodicity of Packet Generation

In this section, we also investigate the performance of mitigation methods with non-periodic packet generation. In particular, the packet generation is assumed to follow the CAM generation rules detailed in [35], which is related in our case to the vehicle speed. Since vehicles are moving at different speeds, the generation frequency is different from vehicle to vehicle, and always lower than the periodicity of resource allocation in NR-V2X (which is 10 Hz).

In the case of IEEE 802.11p and LTE-V2X, which are both shown in [5,12], a periodic generation with a frequency equal to the resource allocation periodicity of LTE-V2X allows for improving the performance of the No-method test and some of the mitigation methods. This effect is a consequence of the LTE-V2X sensing procedure, which looks at the power measured in the last 1 s to estimate how the resources will be used in the future.

Different from LTE-V2X, where the estimation of the resources that should not be used is mainly based on the average sensed power, in NR-V2X it is mainly based on the control information associated with the decoded messages (see, e.g., [15,26] for details). This implies that the periodical transmissions in IEEE 802.11p are ignored by NR-V2X and therefore do not help to reduce the performance worsening due to co-channel coexistence.

**Figure 15:**
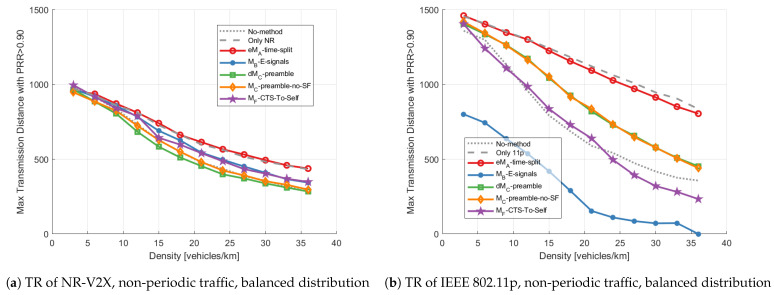
Transmission range vs. density applying different mitigation methods with balanced distribution and non-periodic packet generation: (**a**) shows the TR of NR-V2X, (**b**) shows the TR of IEEE 802.11p.

These considerations are confirmed by Figure 15, where the TR of both technologies with non-periodic packet generation and balanced technology distribution is shown. Comparing the results in Figure 15 with those of Figure 10, it can be noted that the packet generation pattern has little impact on all mitigation methods. This conclusion is also valid for the other metrics and the unbalanced technology distribution, although results are not shown for the sake of conciseness.

## 5. Conclusions

In this paper, we focused on co-channel coexistence between IEEE 802.11p and 5G NR-V2X to investigate the reciprocal interference and the impact of possible mitigation methods. In particular, we evaluated the performance of a number of mitigation methods, here called e$M_{A}$-time-split, $M_{B}$-E-signals, d$M_{C}$-preamble, $M_{C}$-preamble-no-SF, and $M_{F}$-CTS-to-Self, which have been recently proposed by the ETSI with a focus on ITS-G5 and LTE-V2X. Simulations have been carried out through the open-source simulator WiLabV2Xsim. In addition to a summary of the methods and a discussion of their applicability to NR-V2X, results were provided in highway scenarios in terms of various metrics, considering variable vehicle density, technology distribution, and data generation patterns.

The main conclusions from the perspective of both NR-V2X and IEEE 802.11p are summarized in Table 5. As summarized, with a relatively low-density scenario, only e$M_{A}$-time-split and d$M_{C}$-preamble/$M_{C}$-preamble-no-SF are able to improve the transmission range of one technology without reducing the performance of the other, which is in line with the conclusion reported for LTE-V2X and IEEE 802.11p in [4]. The results in this paper show that e$M_{A}$-time-split, which requires modifications only to IEEE 802.11p, performs best in terms of the transmission range in most of the cases for both technologies; the transmission range was shown to reduce significantly only for IEEE 802.11p under high-density conditions when the technology distribution is unbalanced towards the same technology. e$M_{A}$-time-split was also shown to increase the end-to-end delay of IEEE 802.11p compared with the No-method tests (no mitigation method applied), which might be a problem for the Day-2 delay-sensitive applications.

$M_{B}$-E-signals and $M_{F}$-CTS-to-Self, which require modifications in both the technologies, were shown to improve the transmission range of NR-V2X if there are relatively enough time resources (i.e., with the exception of unbalanced traffic with more NR-V2X stations), although they reduce the transmission range of IEEE 802.11p in most cases and also increase the delay of IEEE 802.11p compared with the No-method scenario.

Finally, d$M_{C}$-preamble and $M_{C}$-preamble-no-SF, which require modifications only to NR-V2X (and are therefore applicable also in Europe, where vehicles equipped with ITS-G5 are already being registered) were shown to improve the transmission range of IEEE 802.11p, without negatively affecting the other metrics compared with the No-method results in either technology. Between the two, $M_{C}$-preamble-no-SF provided slightly better performance than the d$M_{C}$-preamble in some cases, despite its simpler implementation.

When comparing these results to those obtained focusing on the coexistence between IEEE 802.11p and LTE-V2X, most of the conclusions are very similar. However, two differences can be remarked. The first one is related to the numerology; when the numerology is different from the one adopted in LTE-V2X, meaning that the subcarrier spacing is larger and the time transmission interval is shorter, there are mitigation methods where adjustments are needed before they are applicable (e.g., d$M_{C}$-preamble). The second one is that periodic and non-periodic traffic bring with NR-V2X similar results; this effect, confirming that the packet generation pattern has little impact on all the mitigation methods when NR-V2X is assumed, is different from what was observed on the coexistence of IEEE 802.11p with LTE-V2X; as explained in this document, this is due to the different sensing and reservation mechanism.

It is relevant to note that the conclusions derived in this paper can also be applied to the co-channel coexistence of IEEE 802.11bd and NR-V2X. In fact: (i) IEEE 802.11bd and IEEE 802.11p share the same channel access scheme and preamble; and (ii) if IEEE 802.11bd applies a similar coding rate to IEEE 802.11p, they have extremely comparable packet durations, which would not modify the impact on NR-V2X and may only imply a slight improvement of the performance of the IEEE technology. Additional studies may be needed only when specific features are applied, such as the repetitions or the channel bonding.

Among the main aspects that may deserve attention in future work are the impact of the variable packet size and the congestion control mechanisms, which may require a redesign to guarantee fair access to the shared medium.

## Figures and Tables

**Table 1:** Investigated co-channel coexistence mitigation methods.

Method Name	Letter	Modifications to NR-V2X	Modifications to 11p	Known Superframe	Limitation
$M_{A}$-time-split	A	None	Need to align to the superframe; Delay transmission artificially	NR-V2X and 11p	Need high-level synchronization
$M_{B}$-E-signals	B	Sending 3 types of energy signals	Reducing CCA threshold from −65 dBm to −85 dBm	NR-V2X	More energy consumption at $V_{\mathrm{NR}}$
$M_{C}$-preamble	C	IEEE 802.11p PHY header insertion and technology proportion estimation	None	NR-V2X	With numerology 2 reduces NR-V2X efficiency; $V_{11p}$ unaware of slots
$M_{C}$-preamble-no-SF	(variant)	Only IEEE 802.11p PHY header insertion	None	None	With numerology 2 reduces NR-V2X efficiency
$M_{F}$-CTS-to-Self	F	Add CTS-to-Self message at the beginning of $S_{\mathrm{NR}}$	Be able to recognize the CTS-to-Self message and set the NAV accordingly	NR-V2X	CTS-to-Self messages may collide; complexity is added to NR-V2X

**Table 2:** Comparison between messages required by different methods.

Message	Required Methods	Characteristics
Energy signal	$M_{B}$-E-signals	• Energy without information • Transmitted in all of the idle intervals in the time domain of $S_{\mathrm{NR}}$
11p-preamble	$M_{C}$-preamble; $M_{E}$-reservation-&-preamble	• Not really a message, but only a portion of signal which is always identical; can be implemented as a fixed sequence of IQ samples • Appended just before each NR-V2X packet
Reservation	$M_{D}$-reservation; $M_{E}$-reservation-&-preamble	• 11p-type message with CSMA/CA channel access protocol • Sent before each NR-V2X packet
CTS-to-Self	$M_{F}$-CTS-to-Self	• 11p-type message without CSMA/CA channel access protocol • Different from transmitter to transmitter • Sent at the beginning of each $S_{\mathrm{NR}}$ by selected $V_{\mathrm{NR}}$

**Table 3:** Options for the numerology of 5G NR-V2X in FR 1 and number of symbols required to allocate the IEEE 802.11p preamble.

Numerology	TTI Duration [µs]	$T_{\mathrm{OFDM}}$ [µs]	Fitting Preamble	v$2\times T_{\mathrm{OFDM}}$ [µs]	Fitting Preamble
0	1000	71.4	✓	142.9	✓
1	500	35.7	✗	71.4	✓
2	250	17.9	✗	35.7	✗

**Table 4:** Main simulation parameters and settings.

**Common Settings**	
Scenario	Highway, 3 + 3 lanes, variable vehicle density, average speed 120 km/h with 12 km/h std. deviation
Data traffic	Packets with 350 bytes payload generated periodically every 100 ms or following the CAM generation rules
Channel and power	Single 10 MHz channel at 5.9 GHz, Tx power 23 dBm (not including antenna gain), antenna gain 3 dBi, noise Figure 6 dB
Propagation	Modified ECC Report 68 rural [27] path-loss model, log-normal shadowing with 3 dB variance and decorr. dist. 25 m
Other settings	Ideal synchronization, congestion control disabled
**For IEEE 802.11p**	
MAC settings	Contention window 15, AIFS 110 µs, SIFS 32 µs
MCS-11p Rx thresholds	−65 dBm with unknown signals; −85 dBm with known signals or when $M_{B}$-E-signals is assumed
**For NR-V2X**	
MCS-NR	MCS 8 (QPSK, CR≈0.60), with SINR threshold 7.7 dB [15] Each packet (350 bytes) occupies 2 subchannels
PHY layer	SCS 30 kHz, 12 physical resource blocks (PRBs) in each subchannel, which means 2 subchannels in the 10 MHz channelThe first-stage sidelink control information (SCI) occupies 3 OFDM symbols and 12 PRBs18 resource elements (REs) are used as demodulation reference signal (DMRS) during each TTIBlind retransmissions are disabled
**Coexistence**	
Superframe	$T_{\mathrm{SF}}=25$ ms, $T_{\mathrm{NR}}=13$ ms, $T_{11p/\mathrm{bd}}=12$ ms
Technology Ratio	Balanced ($V_{\mathrm{NR}}$:$V_{11p}$ = 1:1) or unbalanced with More-$V_{11p}$ ($V_{\mathrm{NR}}$:$V_{11p}$ = 1:2) or unbalanced with More-$V_{\mathrm{NR}}$ ($V_{\mathrm{NR}}$:$V_{11p}$ = 2:1)

**Table 5:** Comparison of the performance between each method and the No-method test.

Method	Transmission Range	End-to-End Delay	Data Age
e$M_{A}$-time-split	NR-V2X: ↑↑, ↑↑, ↑ 11p: ↑↑, ↓(high dens.), ↑↑	NR-V2X: =, =, = 11p: ↓↓, ↓↓, ↓↓	NR-V2X: ↑↑, ↑↑, ↑ 11p: ↓, ↓, ↓
$M_{B}$-E-signals	NR-V2X: ↑, ↑, ↓ 11p: ↓, ↓↓, ↓	NR-V2X: =, =, = 11p: ↓, ↓, ↓	NR-V2X: ↑, ↑, ↑ 11p: ↓↓, ↓↓, ↓↓
d$M_{C}$-preamble & $M_{C}$-preamble-no-SF	NR-V2X: =, =, = 11p: ↑, ↑, ↑	NR-V2X: =, =, = 11p: =, =, =	NR-V2X: =, =, = 11p: =, =, =
$M_{F}$-CTS-to-Self	NR-V2X: ↑, ↑, ↓ 11p: ↓(high dens.), ↓, =(high dens.)	NR-V2X: =, =, = 11p: ↓, ↓, ↓	NR-V2X: ↑, ↑, ↑ 11p: ↓, ↓, ↓

**NOTE:** Each metric contains three arrows corresponding to three scenarios: balanced traffic, more-$V_{11p}$, and more-$V_{\mathrm{NR}}$. ↑ performance improved (larger TR, lower EED, lower DA), similar performance, ↓ performance decreased. Two arrows indicate that the improvement or worsening is significant.

## Data Availability

The simulator with the mitigation methods will be published in its next release and the related simulation code and/or results will be available at https://github.com/V2Xgithub/WiLabV2Xsim/tree/main/codeForPaper/Zhuofei2023cochannel (accessed on 25 April 2023).
